# Type 2 Diabetes Mellitus and its comorbidity, Alzheimer’s disease: Identifying critical microRNA using machine learning

**DOI:** 10.3389/fendo.2022.1084656

**Published:** 2023-01-19

**Authors:** Hind Alamro, Vladan Bajic, Mirjana T. Macvanin, Esma R. Isenovic, Takashi Gojobori, Magbubah Essack, Xin Gao

**Affiliations:** ^1^ Computer, Electrical and Mathematical Sciences and Engineering Division (CEMSE), King Abdullah University of Science and Technology (KAUST), Thuwal, Saudi Arabia; ^2^ Computational Bioscience Research Center (CBRC), King Abdullah University of Science and Technology, Thuwal, Saudi Arabia; ^3^ College of Computer and Information Systems, Umm Al-Qura University, Makkah, Saudi Arabia; ^4^ Department of Radiology and Molecular Genetics, VINCA Institute of Nuclear Science - National Institute of the Republic of Serbia, University of Belgrade, Belgrade, Serbia

**Keywords:** Alzheimer’s disease, biomarker, diabetes, comorbidity, microRNA, machine learning, hsa-mir-103a-3p, hsa-mir-124-3p

## Abstract

MicroRNAs (miRNAs) are critical regulators of gene expression in healthy and diseased states, and numerous studies have established their tremendous potential as a tool for improving the diagnosis of Type 2 Diabetes Mellitus (T2D) and its comorbidities. In this regard, we computationally identify novel top-ranked hub miRNAs that might be involved in T2D. We accomplish this *via* two strategies: 1) by ranking miRNAs based on the number of T2D differentially expressed genes (DEGs) they target, and 2) using only the common DEGs between T2D and its comorbidity, Alzheimer’s disease (AD) to predict and rank miRNA. Then classifier models are built using the DEGs targeted by each miRNA as features. Here, we show the T2D DEGs targeted by hsa-mir-1-3p, hsa-mir-16-5p, hsa-mir-124-3p, hsa-mir-34a-5p, hsa-let-7b-5p, hsa-mir-155-5p, hsa-mir-107, hsa-mir-27a-3p, hsa-mir-129-2-3p, and hsa-mir-146a-5p are capable of distinguishing T2D samples from the controls, which serves as a measure of confidence in the miRNAs’ potential role in T2D progression. Moreover, for the second strategy, we show other critical miRNAs can be made apparent through the disease’s comorbidities, and in this case, overall, the hsa-mir-103a-3p models work well for all the datasets, especially in T2D, while the hsa-mir-124-3p models achieved the best scores for the AD datasets. To the best of our knowledge, this is the first study that used predicted miRNAs to determine the features that can separate the diseased samples (T2D or AD) from the normal ones, instead of using conventional non-biology-based feature selection methods.

## 1 Introduction

Diabetes mellitus affects approximately 463 million adults worldwide, and it is predicted that 700 million individuals will be affected by 2045, per the 2019 International Diabetes Federation report (1). Thus, many research studies are aimed toward Diabetes prevention and/or treatment. Also, the ADDITION-Europe Simulation Model Study shows early diagnosis reduces the risk of suffering cardiovascular events and mortality (2), which also spurs research associated with early diagnosis.

Diabetes mellitus is a metabolic disease characterized by hyperglycemia. In such cases, hyperglycemia results from defects in insulin secretion and/or insulin action (3). Over 90% of the Diabetes mellitus cases are Type 2 Diabetes (T2D) that results from an insulin action defect, i.e., insulin resistance (4). Pancreatic endocrine islet β-cells create and release the two crucial hormones that regulate blood glucose levels: insulin, which acts to lower blood sugar, and glucagon, which raises blood sugar. Thus, the dysfunction of pancreatic islet β-cells is a significant cause of T2D.

MiRNAs play a pivotal role in the regulation of gene expression and are estimated to regulate over 60% of all human genes (5). The miRNA sequence being complementary to the 3′UTR of its target mRNA determines the regulatory effect of miRNA (6). The miRNA associating with its target mRNA can result in translational repression, mRNA deadenylation, or mRNA cleavage (7). Several research findings demonstrate the role of miRNAs in β-cell stimulus–secretion coupling and insulin biosynthesis. For example, miR-15a, miR-24, miR-26, miR-30d, miR-122, miR-127, miR-133, miR-148, miR-182, miR-184, miR-200, miR-204, and miR-375 have demonstrated involvement in insulin biosynthesis (8). Additionally, miR-7, miR-9, miR-29a, miR-96, miR-124, miR-335, and miR-375 were involved in the exocytotic process. More recently, several of these miRNAs and others were experimentally shown to be involved in T2D. For example, Sun and colleagues demonstrated β-cell-specific transgenic *miR-29a/b/c* mice fed a high-fat diet (HFD) are predisposed to develop insulin resistance and glucose intolerance (9). Moreover, they show blocking *miR-29* effects attenuates inflammation and T2D, which supports the findings reported by (10). Su and colleagues determined the miRNAs expression profiles in the pancreas of high-fat diet (HFD) fed Zucker diabetic fatty (ZDF) rats and ‘normal’ Zucker lean (ZL) rats and identified 24 differentially expressed miRNAs among which *miR-34a-5p* and miR-452-5p were the most significantly up- and down-regulated, respectively (11). In Addition, Liu and colleagues demonstrated that overexpression of *miR-296-5p* suppressed β-cells proliferation, arrested cell cycle progression, and increased the healing rate of diabetic wounds both *in vivo* and *in vitro*. Moreover, they provide TargetScan analysis that shows miR-296-5p is a direct regulator of sodium-glucose cotransporter 2 (SGLT2) gene, which is significant as SGLT2 inhibitors have shown promise in diabetes therapy (12).

Moreover, miRNAs are most suitable as biomarkers as they are stable in biofluids such as serum, plasma, blood, tears, urine, or saliva, collected in a minimally invasive manner, even after several freeze-thaw cycles (13, 14). In this regard (15), propose serum miR-491-5p, miR-1307-3p and (16) propose serum as potential biomarkers for diagnosis of pre-diabetes and T2D.

Patients with T2D have higher risks of developing comorbidities which include cardiovascular complications (17), hypertension (17, 18), depression (17–19) thyroid gland diseases (20), chronic obstructive pulmonary disease (COPD) (21), Alzheimer’s disease (AD) (22), amongst others. The existence of these comorbidities means the intersection between genes expressed in T2D and its comorbidity and the genes-miRNA relationships are important to the disease’s progression. In this regard, Pescador and colleagues identified serum miR-15b, miR-138, and miR-376a as having predictive value for T2D and obesity (22, 23), and Seleem and colleagues identified serum miR-342 and miR-450 as indicators of coronary artery disease in T2D patients (24). Luo and colleagues also identified circulating miR-30c as a predictive biomarker of T2D with coronary heart disease (25). No such study exists for T2D and AD even though several miRNAs we mention here, including miR-9, miR-124, miR-127, and miR-200, linked to T2D progression, has also been identified as differentially expressed in AD (26), and several AD gene expression datasets are freely available.

Thus, our study is directed towards computationally identifying novel top-ranked hub miRNAs that might be involved in T2D. We accomplish this *via* two strategies, 1) by ranking miRNAs based on the number of T2D DEGs they target, and 2) using only the common DEGs between T2D and its comorbidity, AD. For the first strategy, the miRNAs are ranked based on the number of T2D DEGs they target. Then T2D classifier models are built using the DEGs targeted by each miRNA as features. Here, we use the feature’s ability to distinguish T2D samples from the control samples as a measure of confidence in the miRNAs’ potential role in T2D progression. For the second strategy, we repeat this process using only the common DEGs between T2D and AD to identify miRNAs capable of distinguishing T2D samples from control samples and AD samples from control samples.

## 2 Materials and method

### 2.1 Gene expression data

To find gene expression datasets of T2D patients, we searched the Gene Expression Omnibus (GEO) database (27) using the query: “Type 2 Diabetes* AND Homo sapiens” filtered by “Expression profiling by array” on the 5th of October 2022. As a result, we retrieved 147 entries, from which we selected four datasets, GSE76895 (28), GSE76894 (29), GSE25724 (30), and GSE20966 (31), used in this study. The GSE76895 dataset comprises 68 samples (36 test and 32 control), GSE76894 103 samples (19 test and 84 control), GSE25724 13 samples (6 test and 7 control), and GSE20966 20 samples (10 test and 10 control) (see **Table 1**).

**Table 1:** Description of the GEO gene expression datasets.

Dataset IDs	Disease	Region	Healthy control	Test	Female/Male
GSE76895	T2D	Human pancreatic islets	32	36	29/39
GSE76894	T2D	Human pancreatic islets	84	19	52/51
GSE25724	T2D	Human pancreatic islets	6	7	6/7
GSE20966	T2D	Human pancreatic islets	10	10	7/13
GSE5281	AD	Entorhinal cortex, Hippocampus, Medial temporal gyrus, Posterior cingulate, Superior frontal gyrus, Primary visual cortex	87	74	58/103
GSE48350	AD	Hippocampus, Entorhinal cortex, The superior frontal cortex, Post-central gyrus	80	173	129/124
GSE1297	AD	Hippocampus, Entorhinal cortex.	22	9	18/13

We also used GEO to find the AD datasets but used the query: “Alzheimer* AND Homo sapiens” filtered by “Expression profiling by array” on the 2nd March 2022. We retrieved 188 entries which we sifted through. We found three gene expression datasets (GSE5281 (32), GSE48350 (33), and GSE1297 (34)) from AD patients and healthy controls within the same age range, generated using the same platform.

### 2.2 Meta-analysis of the gene expression data

To increase the sample size and statistical power, we used Integrative Meta-Analysis of GEO Data (ImaGEO) (35), a web-based platform, to integrate and perform meta-analyses of multiple GEO datasets. We used ImaGEO’s fixed-effect model parameter, with an adjusted p-value < 0.05, and only 10% missing values allowed. Specifically, we used ImaGEO to integrate the GEO T2D datasets (GSE76895, GSE76894), perform background correction, normalization, batch effect correction, and apply initial differential expression analysis. Through this process, ImaGEO generated an integrated matrix with 1918 genes as the potential DEGs, which we used in subsequent analyses. We implemented the same procedure for the AD GEO datasets (GSE5281, GSE48350, and GSE1297), through which we identified 924 DEGs.

Finally, we shortlisted 146 genes that were common between the 1918 T2D DEGs and the 924 AD DEGs.

To further determine the key set of miRNAs associated with the 1918 T2D genes and the 146 common genes, we used miRNet (36). We used multiple settings for miRNet with the 1918 genes, including selecting ‘Homo sapiens’, ‘Official gene symbol’, and ‘miRNA’. Through this process, 1801 genes were mapped to 2640 miRNAs. We then used the ‘Degree Filter’ setting to apply a degree cutoff of 475.0 to the ‘miRNA nodes only’ setting, to restrict our search to the 10 ≥ top-ranked hub miRNAs.

Here it should be noted that miRNA-target mRNA relationships are best established through time-consuming and expensive wet-lab experiments. However, this is an infeasible approach since miRNAs have numerous target genes. Thus, several computational methods that predict miRNA target interactions have been developed based on a combination of different characteristics, including sequence complementarity, evolutionary conservation (37–39), free energy (40,) (41), and/or target site accessibility (42). Examples of popular tools that have been developed using Machine learning (ML) with these characteristics include, TargetScan (43), miRanda (44), PITA (42), and amongst others (26, 45). The miRNA target genes interaction identified through these tools and the functional studies of miRNAs using high-throughput experimental technologies produced an extensive amount of high-quality data regarding miRNA and their target genes that are difficult to sift through. Fortunately, miRNet provided an easy-to-use web-based tool that offers statistical, visual, and network-based approaches to deal with the comprehensive miRNA networks which we use in this study. miRNet provide access to miRNA-target interaction data from well-annotated databases, including miRTarBase (46), miRecords (47), miRanda (44), EpimiR (48), TarBase (49), SM2miR (50), Pharmaco-miR (51), miR2Disease (52), PhenomiR (53), StarBase (54), and miRDB (55).

### 2.3 Developing ML models

To evaluate the ability of the genes to distinguish between the test samples and the healthy controls, we implemented two ML classification models, specifically Random Forests (RF) and Adaboost (AB). We implemented RF and AB models using Python programming language and the Scikit-learn Python library (56).

For the ML models, we created a search space for parameter optimization; we used the GridSearchCV algorithm from Scikit-learn for the hyperparameter optimization. Moreover, since the data samples in both classes are imbalanced, we oversampled the minority class data samples in the training data using Synthetic Minority Oversampling Technique (SMOTE). We implemented the oversampling process using the imblearn python package (57).

To create the feature matrices needed to train the ML models, we downloaded the matrix file provided by GEO for each dataset (GSE76895 and GSE76894). Then, we integrated the samples of the two datasets using R. We had the full matrix containing 171 samples and 1918 features. The features of this dataset are the gene expression profiles of the DEGs identified in this study. After that, for each experiment, we selected from these DEG features as required in experiments (e.g., DEGs associated with each top-ranked miRNA or DEGs common to T2D and AD datasets associated with each top-ranked miRNA). The labels of our dataset are 0 if the sample is healthy and 1 if the sample is T2D.

We fed our feature matrices into ML models. To evaluate these models, we used cross-validation methods with 5 folds. Specifically, we did not separate the datasets into training and testing. Instead, in the training part, we used the combined GSE76895 and GSE76894 datasets and implemented the five-fold cross-validation (CV) technique, which divides the data into 5 subsets. Each subset includes the same percentage of positive and negative samples (i.e., Diabetes and healthy controls). The five-fold cross-validation (CV) technique holds one subset for validation and the other four for training. This process is repeated 5 times to ensure that each subset is used once in the validation part. This process ensures the training data is not mixed with the validation data.

Based on the parameter optimization, in RF, we set the parameters to (max_depth= 10 and n_estimators= 100), and in AB, we used DecisionTreeClassifier as the base estimator and set the parameters to (n_estimators = 200 and learning_rate = 0.001). Finally, we reported the results as area under the curve (AUC) scores. For interpretation of the AUC scores, the closer the value of AUC is to 1, the better the performance.

Also, we employed a secondary testing stage in which we used several external/independent sets, which tests the robustness of the model.

## 3 Results and discussion

### 3.1 The study design

**Figure 1:**
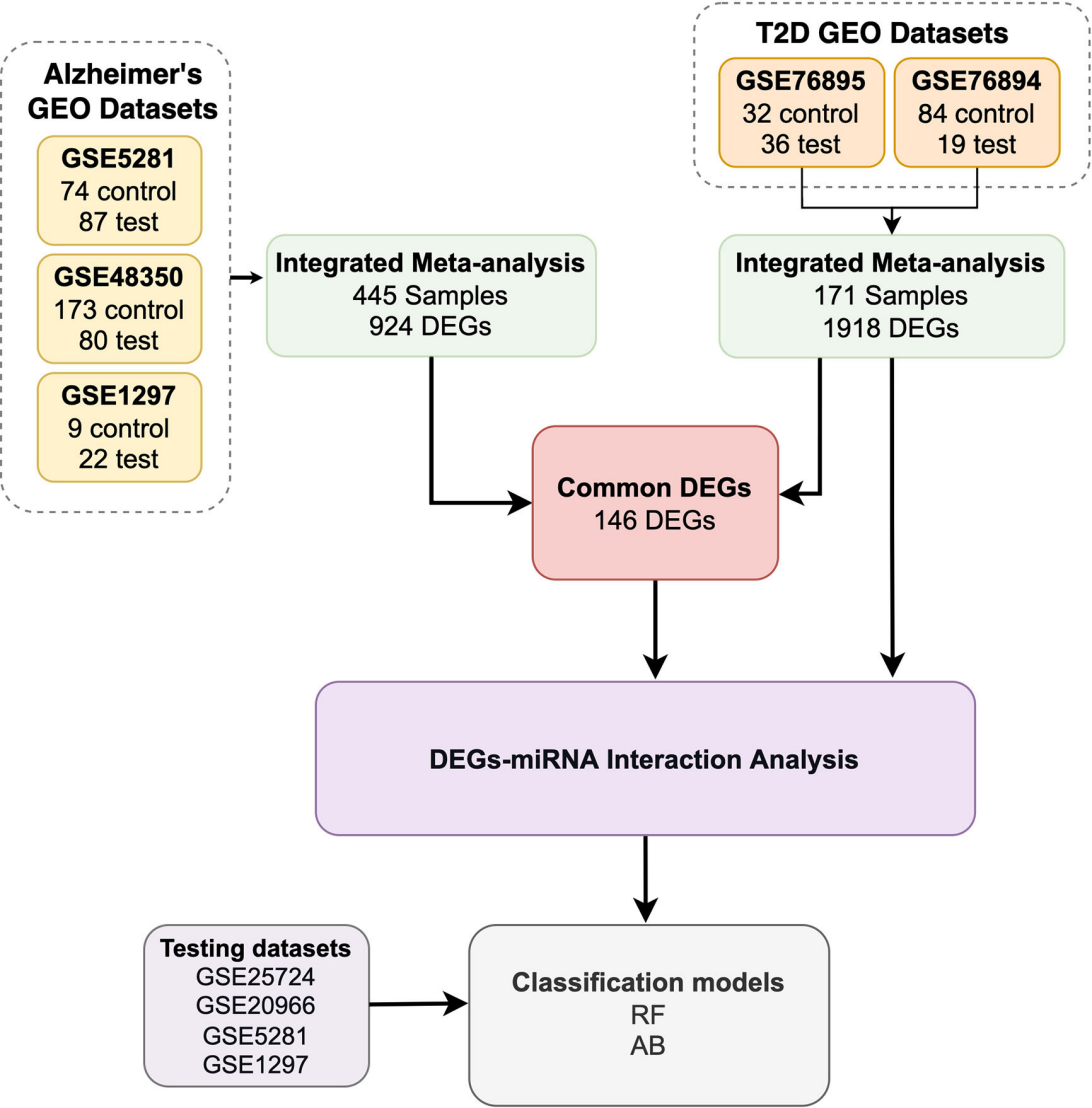
A flowchart description of the study design.

The workflow of our study includes five steps, see **Figure 1**:

1. We retrieved seven GEO datasets. In total, we obtained 445 AD samples and 325 T2D samples.
2. We used 171 of the 325 T2D samples to determine T2D DEGs and all 445 AD samples to determine the AD DEGs. We identified the common DEGs between the T2D and AD samples (146 common DEGs).
3. We utilized the miRNet tool to identify the miRNAs that target the T2D DEGs and the 146 common DEGs.
4. We developed and evaluated ML models using the DEGs determined in the previous steps.

We discuss the steps in detail in the corresponding subsections.

### 3.2 Using miRNA targets to define the gene sets used in evaluating ML models

In the first part of our models’ framework, we used GEO datasets (GSE76895 and GSE76894) to determine T2D DEGs. Then, instead of using conventional non-biology-based feature selection methods such as LASSO regression (22) and Ridge regression (23) to select the gene set/features that would provide optimal prediction performance, we predefined the sets of features as each hub miRNA’s targets.

The non-biology-based feature selection methods refer to the conventional ML feature selection methods. These methods rank the features according to their ability to predict the correct class. That is, it allows for the top 10 or 20 ranked features to be selected to build a model that can best distinguish between disease samples and the controls without considering biological levels of control. However, in our study, we don’t use the ML feature selection methods to define the best set of features; instead, we predefined the sets of features as each hub miRNA’s targets. In this way, we compare the performance of different sets of miRNA-mapped DEGs. Therefore, if a set can distinguish between disease and control samples and produce good results, it indicates the importance of the DEGs and miRNA in the disease state.

**Figure 2:**
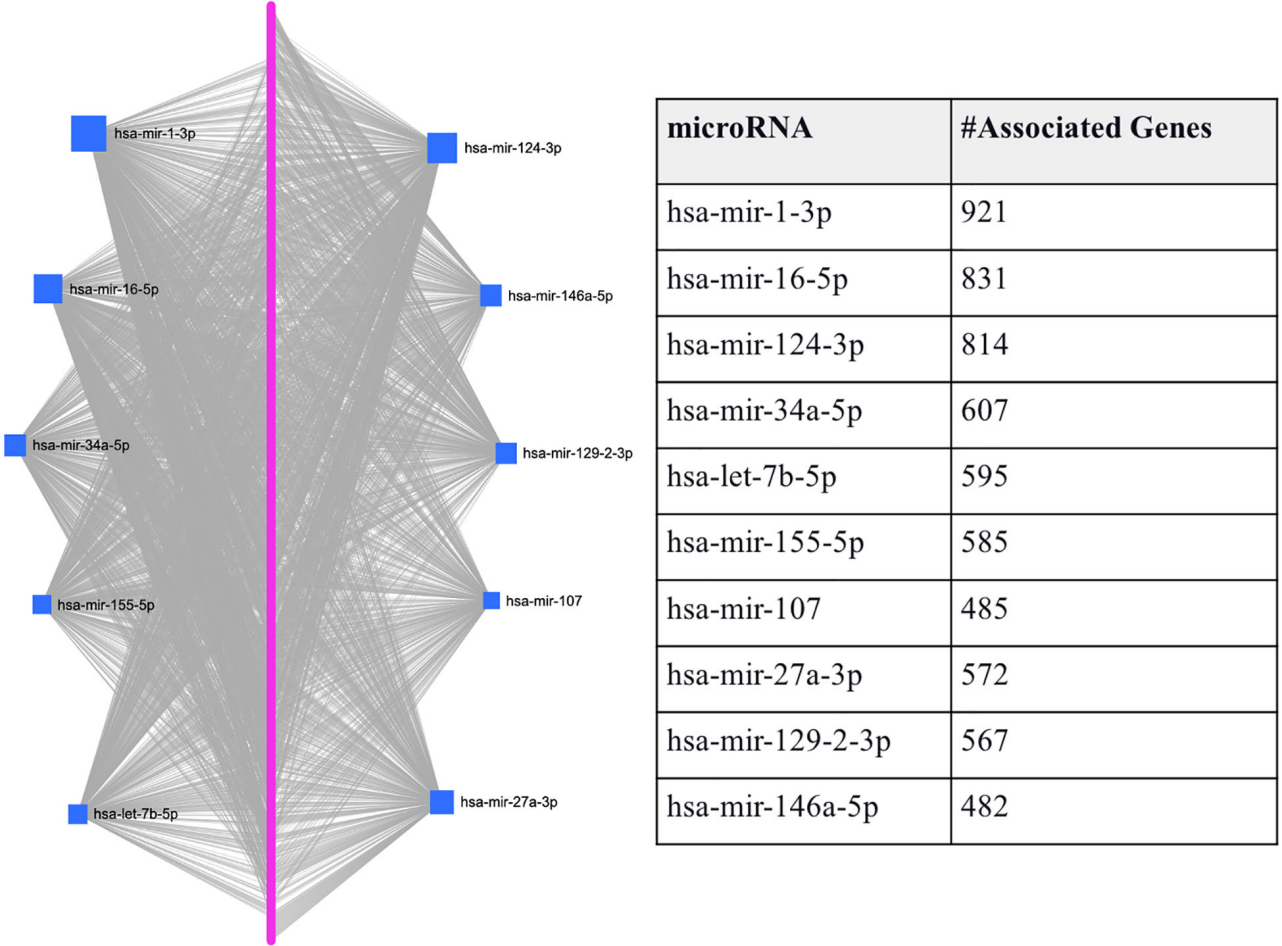
Graphical representation of the top-ranked miRNAs predicted to target T2D DEGs. The pink dots arranged linearly are a series of nodes representing the DEGs targeted by the miRNAs, while the blue squares indicate the miRNAs. The size of the square indicates the degree (the number of targeted genes); that is, the bigger the number of genes targeted by the miRNA, the bigger the size of the square.

To predefine the sets of features as each hub miRNA’s targets, we predicted the miRNAs that target the 1918 T2D DEGs using miRNet. Through this process, we identified 10 miRNAs (hsa-mir-1-3p, hsa-mir-16-5p, hsa-mir-124-3p, hsa-mir-34a-5p, hsa-let-7b-5p, hsa-mir-155-5p, hsa-mir-107, hsa-mir-27a-3p, hsa-mir-129-2-3p, and hsa-mir-146a-5p) that are associated with the majority of T2D DEGs based on miRNet (see **Figure 2**). **Figure 2** shows that each miRNA is predicted to affect about 400 - 950 genes, which we use individually as a gene set/features, similar to features determined by the conventional feature selection method. This method of determining features produces a larger set of features than the conventional feature selection methods (24, 25), which may affect the prediction accuracy achieved by the ML model. However, achieving optimal prediction performance is not the goal here, but rather to use the ML model to gauge if we can use biology in the form of miRNA targets to determine the gene set/s that not only allows good classification of T2D and healthy samples but also allow determining genes-miRNAs relationships that are potentially key determinants in T2D specific functions.

Here, we used the integrated feature matrix (created with GSE76895 and GSE76894) and the submatrices corresponding to the DEGs associated with each of the top-ranked miRNAs to evaluate the DEGs’ ability to distinguish between the T2D sample and the healthy controls. Specifically, we fed the DEGs potentially targeted by each of the ten top-ranked hub miRNAs (see **Figure 2**) to RF and AB classifiers separately as features. For both classification models, we used cross-validation with 5 folds and resampled the minor class using SMOTE to make a balance training set and to avoid bias in the classification. We evaluated the performance of the ML classifiers in each fold based on the area under the curve (AUC) metric.

In **Table 2**, we provide the aggregate results in the form of the average AUC obtained for the five folds. Here it is important to note that we consider AUC results < 0.6 a failure, while AUC results ranging from 0.9-1, 0.8-0.9, 0.7-0.8, and 0.6-0.7 are considered excellent, good, fair, and poor, respectively. Thus, both RF and AB classifiers achieved excellent and good results for all the tested gene sets.

**Table 2:** Prediction performances achieved by RF and AB models using the targeted genes as features.

microRNA	RF	AB	Average
hsa-mir-1-3p	0.915	0.8753	0.89515
hsa-mir-16-5p	0.91	0.8627	0.88635
hsa-mir-124-3p	0.8786	0.9155	0.89705
hsa-mir-34a-5p	0.9005	0.9018	0.90115
hsa-let-7b-5p	0.9167	0.9235	0.9201
hsa-mir-155-5p	0.8562	0.8943	0.87525
hsa-mir-107	0.8991	0.8677	0.8834
hsa-mir-27a-3p	0.9115	0.9349	0.9232
hsa-mir-129-2-3p	0.8983	0.8566	0.87745
hsa-mir-146a-5p	0.8964	0.9133	0.90485
All DEGs	0.9109	0.9138	0.91235

Specifically, for all the gene sets tested, the AB and RF classifiers achieved AUC ranging between 0.85 to 0.93. **Table 2** also provides the average AUC achieved for the two models. The average of the AUC scores for the two classifiers shows that the gene sets associated with hsa-let-7b-5p and hsa-mir-27a-3p allow better classification of the T2D and healthy control samples with average AUC scores of 0.92 for both.

This result is partially supported by Al-Kafaji and colleagues (58), who demonstrated that pre-diabetic individuals exhibited significantly higher miR−1 and miR−133 expression levels than the controls (P<0.05). Moreover, they show that when discriminating pre−diabetic individuals from healthy controls with miR−1 and miR−133, an AUC of 0.813 and 0.810 were achieved, respectively. Also, in other studies, liver miR-34a-5p is shown to be involved in hepatic insulin resistance (IR), which plays a crucial role in the development of T2D (59), and miR-27a-3p was shown to be negatively associated with peripheral insulin sensitivity (60).

Also, using all the genes (1918 T2D DEGs), an average AUC of 0.91 was achieved by both classifiers. Here, to show that the classifiers’ ranking of the essential features is in line with our understanding of the pathological process, we also used the Enrichr (61–63) ‘KEGG 2021 Human’ pathway tool to assess if the higher-ranked features function in a more T2D-specific role than the lower-ranked features. We found the top-200 ranked genes picked up T2D as the most enriched pathway followed by insulin resistance, while the T2D pathway was not picked up as enriched for the genes ranked 200-400, 400-600, 600-800, or the lowest ranked 500 genes (results provided in **Supplementary File S2**). This led us to further compare the performances of the two classifiers using the top-200 and top-50 ranked genes. We found that the RF and AB classifiers achieved AUCs of 0.9577 and 0.9290 for the top-200 ranked genes, respectively. Moreover, the performances of both classifiers improved further using the top-50 ranked genes, i.e., RF achieved an AUC of 0.9620, and AB an AUC of 0.9504. Interestingly, miRNet shows the top-50 ranked RF and AB genes are primarily regulated by all the above-identified hub genes (see **Table 2**), except hsa-mir-155-5p is replaced by hsa-mir-195-5p (see **Supplementary File S2**).

### 3.3 Using the intersection between T2D and AD DEGs to build the ML models

**Figure 3:**
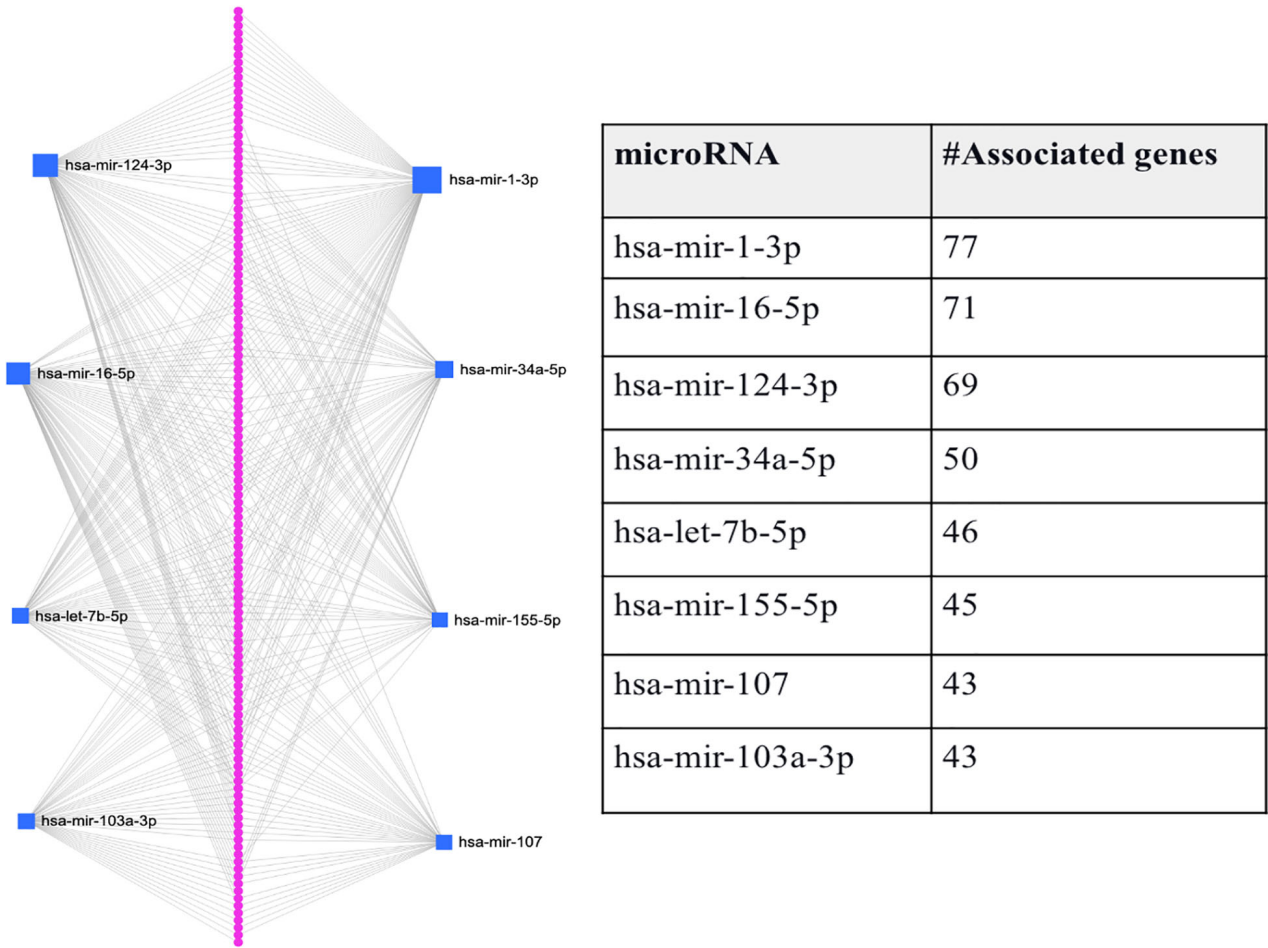
Graphical representation of the top-ranked miRNAs predicted to target the 146 DEGs associated with T2D and its comorbidity, Alzheimer’s disease. The pink dots arranged linearly are a series of nodes representing the DEGs targeted by the miRNAs, while the blue squares indicate the miRNAs. The size of the square indicates the degree (the number of targeted genes); that is, the bigger the number of genes targeted by the miRNA, the bigger the size of the square.

Since comorbidity is common among T2D patients, we here also consider if the intersection between genes expressed in T2D and its comorbidity can also be a means to identify genes-miRNA relationships that are important to the disease’s progression. Here, we consider the intersection between T2D and AD DEGs, as several studies suggest that adults with T2D have a higher risk of developing AD (22). As mentioned above, we identified 146 genes that were common between the 1918 T2D DEGs and the 924 AD DEGs. We predicted the miRNAs that target the 146 genes and found eight miRNAs associated with the majority of genes (hsa-mir-1-3p, hsa-mir-16-5p, hsa-mir-124-3p, hsa-mir-34a-5p, hsa-let-7b-5p, hsa-mir-155-5p, and hsa-mir-103a-3p). **Figure 3** shows that each miRNA is predicted to affect about 43 - 77 genes. Specifically, the first-top miRNA, hsa-mir-1-3p, is associated with 77 of the 146 DEGs, the second-top miRNA, hsa-mir-16-5p, is associated with 71 of the 146 DEGs, and so on. All the miRNAs predicted to target the 146 DEGs, except hsa-mir-103a-3p, were also identified as top-ranked hub miRNAs (see **Figure 3**). Thus, we have used comorbidity here to zoom in on a subset of the genes evaluated in Section 3.2, which may represent a subset of genes essential for T2D and AD progression. **Supplementary File S1** provides the details of genes associated with each miRNA listed in **Figure 3**.

Here, again we used submatrices (from the integrated feature matrix created with GSE76895 and GSE76894) corresponding to the DEGs associated with each of the eight miRNAs and the 146 DEGs. Similar to the process used to evaluate the DEGs’ ability to distinguish between the T2D sample and the healthy controls in section 3.2, we here also fed the DEGs potentially targeted by each of the eight miRNAs to RF and AB classifiers separately as features and used cross-validation with 5 folds for all the classifiers.

**Figure 4:**
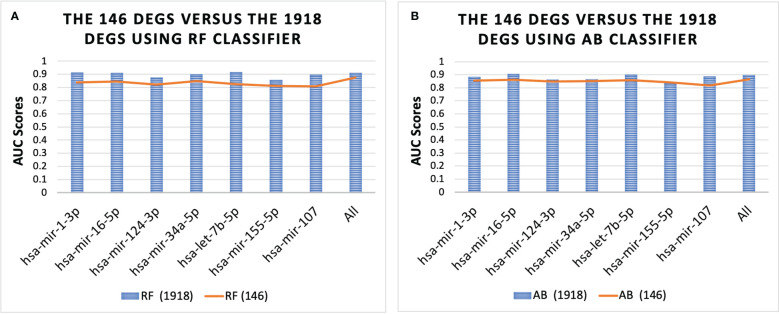
Performance of 146 DEGs and 1918 DEGs using the RF **(A)** and AB **(B)** classifier. The results further show that the DEGs associated with each miRNA could distinguish between the T2D sample and the healthy controls, as they all achieved an average AUC for the classifiers above 0.80. Furthermore, the results for all miRNAs were similar, with average AUC scores ranging from 0.84-0.87, except for the set of genes associated with miRNA hsa-mir-107, which had the lowest scores with AUC = 0.78 for both classifiers.

**Figure 4** shows the performance of the 146 DEGs and 1918 DEGs in RF and AB. The RF and AB classifiers achieved an average AUC score = 0.85 when using the 146 DEGs. Moreover, **Figure 4** shows that even with the huge reduction in the feature size, the performance of the small sets (146 DEGs) is still close to the large sets (1918 DEGs), which suggests that several genes essential for T2D progression were captured in this smaller set. Further, note that hsa-mir-103a-3p is not shown in **Figure 4** because it was not one of the top-ranked miRNAs for the 1918 DEGs, but the genes associated with hsa-mir-103a-3p also achieved an average AUC = 0.84 both the RF and AB classifiers.

### 3.4 Evaluating the performance of the ML classifiers on independent sets

To evaluate the constructed models’ ability to classify the samples, we tested these models using several external/independent testing datasets from T2D and AD. We used the GSE25724 and GSE20966 datasets to evaluate for T2D, and the GSE5281 and GSE1297 datasets for AD. In this experiment, we used all the samples of the combined dataset (GSE76895 and GSE76894) to train the ML models and then tested these models using the external datasets. We evaluated the results by AUC, F1, Precision, and Recall scores. The details of the datasets are provided in **Table 1**.

The GSE5281 dataset contains samples from six brain regions, including Hippocampus (HP), Entorhinal Cortex (EC), Medial Temporal Gyrus (MTG), Posterior Cingulate Cortex (PC), Superior Frontal Gyrus (SFG), and Primary Visual Cortex (VCX). We separated the samples of this dataset according to each region. There are 10 test/13 control samples in GSE5281-HP, 16 test/12 control samples in GSE5281-MTG, 23 test/11 control samples in GSE5281-SFG, and 19 test/12 control samples in GSE5281-VCX.

**Figure 5:**
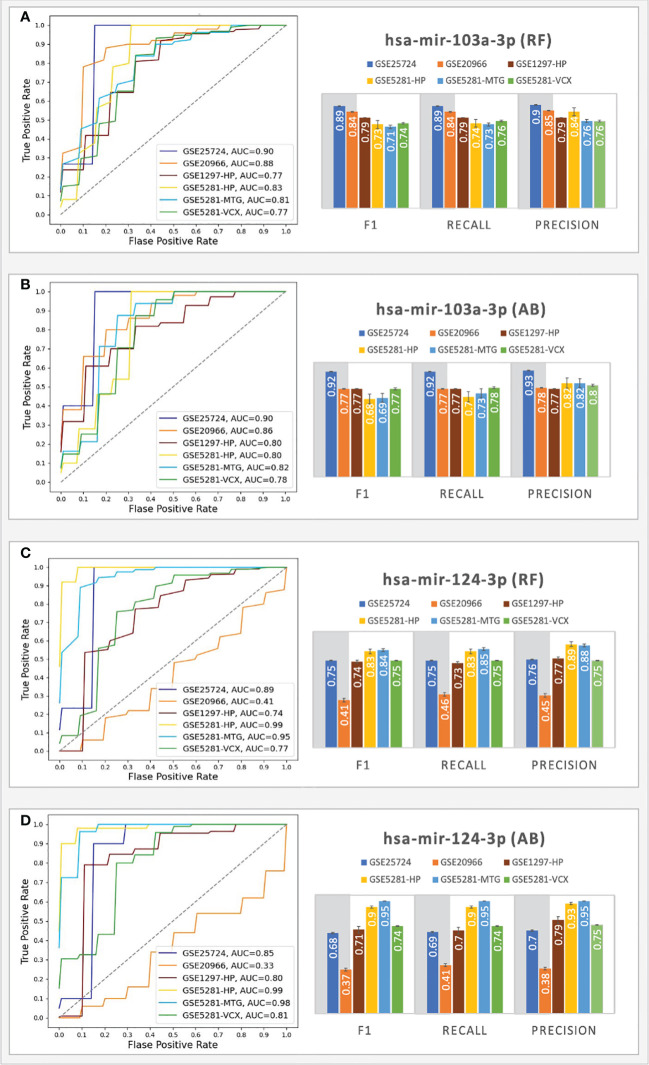
Prediction performances of the ML models (RF, AB) using the genes targeted by hsa-mir-103a-3p **(A, B)** and hsa-mir-124-3p **(C, D)**. The columns in the grey shaded area indicate T2D datasets, while the rest are AD.

For the T2D testing, the RF classifiers achieved the best performance; moreover, the results showed that the genes associated with all the top-ranked miRNAs do not consistently produce good results (F1 scores < 0.70), except for the gene sets associated with hsa-mir-103a-3p and hsa-mir-124-3 (see **Figure 5**).

The RF classifiers achieved AUC = 0.90 (with an F1 score = 0.89) for the GSE25724 dataset and AUC=0.88 (with an F1 score = 0.84) for the GSE20966 dataset. The AB classifiers achieved AUC scores above 0.9 (with an F1 score above 0.77) for the genes associated with hsa-mir-103a-3p, indicating that the 43 genes play important roles in T2D progression and suggesting the involvement of hsa-mir-103a-3p. On the other hand, hsa-mir-124-3p only achieved good performances for the GSE25724 dataset; that is, both classifiers achieved AUCs > 0.85 with F1 scores ranging from 0.68 - 0.75. For the GSE20966 dataset, the genes associated with hsa-mir-124-3p only achieved AUCs ranging from 0.33 - 0.41 and F1 scores ranging between 0.37 - 0.41, which may be a consequence of one missing gene, as Calponin 2 (CNN2) was not picked up as a DEG in the GSE20966 dataset, but it was identified as a DEG in the GSE25724 dataset that achieved AUCs > 0.85. Other datasets did not have any missing genes. Nonetheless, for the T2D datasets, the genes associated with hsa-mir-103a-3p consistently achieved AUC and F1 scores that are on par or better than those associated with hsa-mir-124-3p.

Because we here determined the tested gene sets and miRNAs based on several research studies suggesting that adults with T2D have a higher risk of developing AD (64), we also tested these models using several external testing sets from AD. For the AD testing, the best performances were achieved with the samples taken from the HP, MTG, and VCX region. For these regions, using the genes associated with hsa-mir-103a-3p, the RF classifiers achieved AUC scores ranging from 0.77 - 0.83 (with F1 scores ranging from 0.71 - 0.79), and the AB classifiers achieved AUC scores ranging from 0.78 - 0.82 (with F1 scores ranging from 0.68 - 0.77). Moreover, using the genes associated with hsa-mir-124-3p, the RF classifiers achieved AUC scores ranging from 0.74 - 0.99 (with F1 scores ranging from 0.74 - 0.84), and the AB classifiers achieved AUC scores ranging from 0.80 - 0.99 (with F1 scores ranging from 0.71 - 0.95). The results suggest that the hsa-mir-103a-3p models work well for all the datasets, especially in T2D, while the hsa-mir-124-3p models achieved the best scores for the AD datasets. The results scores of all ML experiments are shown in **Supplementary File S2**. The findings are partially supported by experiments by Zhou and colleagues (65) that show an intracranial injection of miR-124-3p in an AD model mouse significantly reduced amyloid -β protein (Aβ) deposition and improved cognitive outcome.

## 4 Concluding remarks

In this project we identified several top-ranked hub miRNAs (hsa-mir-1-3p, hsa-mir-16-5p, hsa-mir-124-3p, hsa-mir-34a-5p, hsa-let-7b-5p, hsa-mir-155-5p, hsa-mir-107, hsa-mir-27a-3p, hsa-mir-129-2-3p, and hsa-mir-146a-5p) that likely contribute to T2D progression and we used classifiers built using the T2D DEGs targeted by each miRNA to increase confidence in the miRNAs’ potential role in T2D progression. Moreover, the results demonstrate that we can use gene sets targeted by the top-ranked hub genes as features instead of conventional non-biology-based feature selection methods. Moreover, we show other critical miRNAs that can be made apparent through the disease’s comorbidities, in this case, hsa-mir-103a-3p and hsa-mir-124-3p, that we can assess using classifiers before moving to the lab.

Moreover, this study showed several T2D/AD common genes predicted to be targeted by hsa-mir-103a-3p downregulated, including MDH1, PTPN3, POLR2C, MYCN, ACTR3B, UBE2D4, SH2D3C, CYCS, ATXN10, ENO2, XRCC6, RRAGA, BCAS2, MKKS, UBL3, UQCRC2, CCT7, MRPL48, HLF, PARP2, ATP6V0B, MDH2, SNCA, RAD51C, UTP18, MADD, TGFBR3, LAMTOR3, RHBDD3, and NPTX2. Of all these genes, we find NPTX2 to be the most interesting as the Cognitive Vitality Reports (last updated on July 20, 2020) published a piece titled “NPTX2 Modulator” that motivates the need for NPTX2 Modulator. The reason is that NPTX2 (Neuronal Pentraxin 2 gene) becomes increasingly repressed with age (66–68), and decreased NPTX2 levels are associated with cognitive decline, indicating synapse loss, as NPTX2 functions in maintaining synaptic plasticity and inhibitory-excitatory balance in the central nervous system (69–71). Moreover, NPTX2 levels are also reduced in diabetic β-cells, and streptozotocin-damaged islets treated with GLP-1 (Glucagon-like Peptide-1) gene therapy were found to upregulate NPTX2 and the GLP-1 gene therapy exhibit β-cell protective effects (72). However, it is unclear whether or not or how NPTX2 contributes to this β-cell protection.

Here we should note that other diseases, such as Parkinson’s Disease (73–75) and late-stage liver cancer (68, 72, 76) exhibit increased NPTX2 levels; thus, NPTX2 modulators are needed. In addition, there are currently no NPTX2 modulatory drugs, but preclinical efforts are underway to develop NPTX2 modulators. Therefore, in this regard, hsa-mir-103a-3p may be a potential NPTX2 modulator that we need to consider, or maybe an hsa-mir-103a-3p modulator.

Vatandoost and colleagues reported increased miR-103 levels in peripheral blood mononuclear cells from diabetic rats compared to the control group (77). Luo and colleagues further showed that the circulating miR-103 family are potential biomarkers for T2D through targeting genes coding for caveolin 1 (CAV-1) and secreted frizzled-related protein 4 (SFRP4) (61). Interestingly, Trajkovski and colleagues showed in earlier work CAV-1 as a direct target gene of miR-103 (78). They further demonstrated that the insulin receptor regulator, CAV-1, is upregulated upon miR-103 inactivation in adipocytes. In addition, Natarelli and colleagues show that long noncoding RNAs (lncRNAs) are also targets of miR-103. Specifically, they show data that suggest miR-103 programs endothelial cells toward a maladapted phenotype by targeting lncWDR59, which can promote the buildup of atheromatous plaques that causes coronary artery disease, stroke, or kidney problems, depending on the arteries affected. Moreover, miR-103 has been suggested to control voltage-sensitive Ca^2+^ channel expression in brain, thus miR-103 potential role in the exocytosis process through the targeting in genes encoding subunits of voltage-dependent Ca^2+^ channels in beta-cells should also be investigated (79). Moreover, Luo and colleagues (61) demonstrate that circulating miR-103a and miR-103b not only provide high sensitivity and specificity to differentiate the pre-diabetes population but are also T2D biomarkers with high diagnostic value.

These results suggest miR-103 as a potential target for treating T2D, obesity, and cardiovascular diseases, while the current study further suggests miR-103 and hsa-mir-124-3p as potential targets for AD. Beyond this, this work unveiled biomarkers with potential prediction capability towards risk of T2D and (or) AD that we should compare against state-of-art methods in the future when they are publicly available.

## Data availability statement

Publicly available datasets were analyzed in this study. This data can be found here: Gene Expression Omnibus (GEO) database: GSE76895, GSE76894, GSE25724, GSE20966, GSE5281, GSE48350, GSE1297.

## Author contributions

ME, and XG: Conceptualization. VB and HA: Data curation. HA and ME: Methodology. HA: Formal analysis. HA, VB, and EI: Validation. HA, MM, EI, and ME: Writing - original draft. HA, MM, EI, TG, ME, and XG: Writing - review and editing. All authors read and approved the final manuscript.

## References

[1] Saeedi P Petersohn I Salpea P Malanda B Karuranga S Unwin N. Global and regional diabetes prevalence estimates for 2019 and projections for 2030 and 2045: Results from the international diabetes federation diabetes atlas, 9 edition. Diabetes Res Clin Pract (2019) 157:107843. doi: 10.1016/j.diabres.2019.107843 31518657

[2] Herman WH Ye W Griffin SJ Simmons RK Davies MJ Khunti K. Early detection and treatment of type 2 diabetes reduce cardiovascular morbidity and mortality: A simulation of the results of the Anglo-Danish-Dutch study of intensive treatment in people with screen-detected diabetes in primary care (ADDITION-Europe). Diabetes Care (2015) 38:1449–55. doi: 10.2337/dc14-2459 PMC4512138 25986661

[3] American Diabetes A. 2. classification and diagnosis of diabetes: Standards of medical care in diabetes–2020. Diabetes Care (2019) 43:S14–31. doi: 10.2337/dc20-S002 31862745

[4] Jaacks LM Siegel KR Gujral UP Narayan KMV. Type 2 diabetes: A 21st century epidemic. Best Pract Res Clin Endocrinol Metab (2016) 30:331–43. doi: 10.1016/j.beem.2016.05.003 27432069

[5] Hogg DR Harries LW. Human genetic variation and its effect on miRNA biogenesis, activity and function. Biochem Soc Trans (2014) 42:1184–9. doi: 10.1042/BST20140055 25110023

[6] Bhowmick SS Saha I Bhattacharjee D Genovese LM Geraci F. Genome-wide analysis of NGS data to compile cancer-specific panels of miRNA biomarkers. PloS One (2018) 13:e0200353. doi: 10.1371/journal.pone.0200353 30048452 PMC6061989

[7] Ha M Kim VN. Regulation of microRNA biogenesis. Nat Rev Mol Cell Biol (2014) 15:509–24. doi: 10.1038/nrm3838 25027649

[8] Eliasson L Esguerra JLS. Role of non-coding RNAs in pancreatic beta-cell development and physiology. Acta Physiol (2014) 211:273–84. doi: 10.1111/apha.12285 24666639

[9] Sun Y Zhou Y Shi Y Zhang Y Liu K Liang R. Expression of miRNA-29 in pancreatic β cells promotes inflammation and diabetes *via* TRAF3. Cell Rep (2021) 34:108576. doi: 10.1016/j.celrep.2020.108576 33406428

[10] Bagge A Clausen TR Larsen S Ladefoged M Rosenstierne MW Larsen L. MicroRNA-29a is up-regulated in beta-cells by glucose and decreases glucose-stimulated insulin secretion. Biochem Biophys Res Commun (2012) 426:266–72. doi: 10.1016/j.bbrc.2012.08.082 22940552

[11] Su T Hou J Liu T Dai P Qin L Ding L. MiR-34a-5p and miR-452-5p: The novel regulators of pancreatic endocrine dysfunction in diabetic zucker rats? Int J Med Sci (2021) 18:3171–81. doi: 10.7150/ijms.62843 PMC8364455 34400887

[12] Gadzhanova S Pratt N Roughead E. Use of SGLT2 inhibitors for diabetes and risk of infection: Analysis using general practice records from the NPS MedicineWise MedicineInsight program. Diabetes Res Clin Pract (2017) 130:180–5. doi: 10.1016/j.diabres.2017.06.018 28646701

[13] Huang W. MicroRNAs: Biomarkers, diagnostics, and therapeutics. Methods Mol Biol (2017) 1617:57–67. doi: 10.1007/978-1-4939-7046-9_4 28540676

[14] Lu TX Rothenberg ME. MicroRNA. J Allergy Clin Immunol (2018) 141:1202–7. doi: 10.1016/j.jaci.2017.08.034 PMC5889965 29074454

[15] Sidorkiewicz I Niemira M Maliszewska K Erol A Bielska A Szalkowska A. Circulating miRNAs as a predictive biomarker of the progression from prediabetes to diabetes: Outcomes of a 5-year prospective observational study. J Clin Med Res (2020) 9:2184. doi: 10.3390/jcm9072184 PMC7408684 32664305

[16] Yang Z Chen H Si H Li X Ding X Sheng Q. Serum miR-23a, a potential biomarker for diagnosis of pre-diabetes and type 2 diabetes. Acta Diabetol (2014) 51:823–31. doi: 10.1007/s00592-014-0617-8 24981880

[17] Emerging Risk Factors C Sarwar N Gao P Seshasai SRK Gobin R Kaptoge S. Diabetes mellitus, fasting blood glucose concentration, and risk of vascular disease: a collaborative meta-analysis of 102 prospective studies. Lancet (2010) 375:2215–22. doi: 10.1016/S0140-6736(10)60484-9 PMC2904878 20609967

[18] Waeber B Feihl F Ruilope L. Diabetes and hypertension. Blood Press (2001) 10:311–21. doi: 10.1080/080370501753400610 11822535

[19] de Groot M Anderson R Freedland KE Clouse RE Lustman PJ. Association of depression and diabetes complications: a meta-analysis. Psychosom. Med (2001) 63:619–30. doi: 10.1097/00006842-200107000-00015 11485116

[20] Vondra K Vrbikova J Dvorakova K. Thyroid gland diseases in adult patients with diabetes mellitus. Minerva Endocrinol (2005) 30:217–36. 16319810

[21] Feary JR Rodrigues LC Smith CJ Hubbard RB Gibson JE. Prevalence of major comorbidities in subjects with COPD and incidence of myocardial infarction and stroke: A comprehensive analysis using data from primary care. Thorax (2010) 65:956–62. doi: 10.1136/thx.2009.128082 20871122

[22] Kim Y Kim J. Gradient Lasso for Feature Selection. Twenty-first international conference on Machine learning - ICML '04. (2004). doi: 10.1145/1015330.1015364

[23] Zhang S Cheng D Hu R Deng Z. Supervised Feature Selection Algorithm Via Discriminative Ridge Regression. World Wide Web (2018) 21 (6):1545–62. doi: 10.1007/s11280-017-0502-9

[24] Seleem M Shabayek M Ewida HA. MicroRNAs 342 and 450 together with NOX-4 activity and their association with coronary artery disease in diabetes. Diabetes. Metab Res Rev (2019) 35:e3130. doi: 10.1002/dmrr.3130 30681251

[25] Luo M Wang G Xu C Zeng M Lin F Wu J. Circulating miR-30c as a predictive biomarker of type 2 diabetes mellitus with coronary heart disease by regulating PAI-1/VN interactions. Life Sci (2019) 239:117092. doi: 10.1016/j.lfs.2019.117092 31760103

[26] Roy B Lee E Li T Rampersaud M. Role of miRNAs in neurodegeneration: From disease cause to tools of biomarker discovery and therapeutics. Genes (2022) 13:425. doi: 10.3390/genes13030425 35327979 PMC8951370

[27] Clough E Barrett T. The gene expression omnibus database. Methods Mol Biol (2016) 1418:93–110. doi: 10.1007/978-1-4939-3578-9_5 27008011 PMC4944384

[28] Solimena M Schulte AM Marselli L Ehehalt F Richter D Kleeberg M. Systems biology of the IMIDIA biobank from organ donors and pancreatectomised patients defines a novel transcriptomic signature of islets from individuals with type 2 diabetes. Diabetologia (2018) 61:641–57. doi: 10.1007/s00125-017-4500-3 PMC5803296 29185012

[29] Khamis A Canouil M Siddiq A Crouch H Falchi M Bulow MV. Laser capture microdissection of human pancreatic islets reveals novel eQTLs associated with type 2 diabetes. Mol Metab (2019) 24:98–107. doi: 10.1016/j.molmet.2019.03.004 30956117 PMC6531807

[30] Dominguez V Raimondi C Somanath S Bugliani M Loder MK Edling CE. Class II phosphoinositide 3-kinase regulates exocytosis of insulin granules in pancreatic beta cells. J Biol Chem (2011) 286:4216–25. doi: 10.1074/jbc.M110.200295 PMC3039383 21127054

[31] Marselli L Thorne J Dahiya S Sgroi DC Sharma A Bonner-Weir S. Gene expression profiles of beta-cell enriched tissue obtained by laser capture microdissection from subjects with type 2 diabetes. PloS One (2010) 5:e11499. doi: 10.1371/journal.pone.0011499 20644627 PMC2903480

[32] Liang WS Dunckley T Beach TG Grover A Mastroeni D Walker DG. Gene expression profiles in anatomically and functionally distinct regions of the normal aged human brain. Physiol Genomics (2007) 28:311–22. doi: 10.1152/physiolgenomics.00208.2006 PMC2259385 17077275

[33] Berchtold NC Cribbs DH Coleman PD Rogers J Head E Kim R. Gene expression changes in the course of normal brain aging are sexually dimorphic. Proc Natl Acad Sci U. S. A. (2008) 105:15605–10. doi: 10.1073/pnas.0806883105 PMC2563070 18832152

[34] Blalock EM Geddes JW Chen KC Porter NM Markesbery WR Landfield PW. Incipient alzheimer's disease: Microarray correlation analyses reveal major transcriptional and tumor suppressor responses. Proc Natl Acad Sci U. S. A. (2004) 101:2173–8. doi: 10.1073/pnas.0308512100 PMC357071 14769913

[35] Toro-Domínguez D Martorell-Marugán J López-Domínguez R García-Moreno A González-Rumayor V Alarcón-Riquelme ME. ImaGEO: Integrative gene expression meta-analysis from GEO database. Bioinformatics (2019) 35:880–2. doi: 10.1093/bioinformatics/bty721 30137226

[36] Chang L Zhou G Soufan O Xia J. miRNet 2.0: network-based visual analytics for miRNA functional analysis and systems biology. Nucleic Acids Res (2020) 48(W1):W244–W251. doi: 10.1093/nar/gkaa467 PMC7319552 32484539

[37] Lewis BP Burge CB Bartel DP. Conserved seed pairing, often flanked by adenosines, indicates that thousands of human genes are MicroRNA targets. Cell (2005) 120:15–20. doi: 10.1016/j.cell.2004.12.035 15652477

[38] Friedman RC Farh KK-H Burge CB Bartel DP. Most mammalian mRNAs are conserved targets of microRNAs. Genome Res (2009) 19:92–105. doi: 10.1101/gr.082701.108 18955434 PMC2612969

[39] Lewis BP Shih IH Jones-Rhoades MW Bartel DP Burge CB. Prediction of mammalian microRNA targets. Cell (2003) 115:787–98. doi: 10.1016/s0092-8674(03)01018-3 14697198

[40] Krek A Grün D Poy MN Wolf R Rosenberg L Epstein EJ. Combinatorial microRNA target predictions. Nat Genet (2005) 37:495–500. doi: 10.1038/ng1536 15806104

[41] Enright AJ John B Gaul U Tuschl T Sander C Marks DS. MicroRNA targets in drosophila. Genome Biol (2003) 5:R1. doi: 10.1186/gb-2003-5-1-r1 14709173 PMC395733

[42] Kertesz M Iovino N Unnerstall U Gaul U Segal E. The role of site accessibility in microRNA target recognition. Nat Genet (2007) 39:1278–84. doi: 10.1038/ng2135 17893677

[43] Agarwal V Bell GW Nam J-W Bartel DP. Predicting effective microRNA target sites in mammalian mRNAs. eLife (2015) 4:e05005. doi: 10.7554/elife.05005 26267216 PMC4532895

[44] Betel D Koppal A Agius P Sander C Leslie C. Comprehensive modeling of microRNA targets predicts functional non-conserved and non-canonical sites. Genome Biol (2010) 11:R90. doi: 10.1186/gb-2010-11-8-r90 20799968 PMC2945792

[45] Riolo G Cantara S Marzocchi C Ricci C. miRNA targets: From prediction tools to experimental validation. Methods Protoc (2020) 4:1. doi: 10.3390/mps4010001 33374478 PMC7839038

[46] Hsu S-D Lin F-M Wu W-Y Liang C Huang W-C Chan W-L. miRTarBase: a database curates experimentally validated microRNA-target interactions. Nucleic Acids Res (2011) 39:D163–169. doi: 10.1093/nar/gkq1107 PMC3013699 21071411

[47] Xiao F Zuo Z Cai G Kang S Gao X Li T. miRecords: an integrated resource for microRNA-target interactions. Nucleic Acids Res (2009) 37:D105–110. doi: 10.1093/nar/gkn851 PMC2686554 18996891

[48] Dai E Yu X Zhang Y Meng F Wang S Liu X. EpimiR: a database of curated mutual regulation between miRNAs and epigenetic modifications. Database (2014) 2014:bau023. doi: 10.1093/database/bau023 24682734 PMC4037167

[49] Vergoulis T Vlachos IS Alexiou P Georgakilas G Maragkakis M Reczko M. TarBase 6.0: capturing the exponential growth of miRNA targets with experimental support. Nucleic Acids Res (2012) 40:D222–9. doi: 10.1093/nar/gkr1161 PMC3245116 22135297

[50] Liu X Wang S Meng F Wang J Zhang Y Dai E. SM2miR: a database of the experimentally validated small molecules' effects on microRNA expression. Bioinformatics (2013) 29:409–11. doi: 10.1093/bioinformatics/bts698 23220571

[51] Rukov JL Wilentzik R Jaffe I Vinther J Shomron N. Pharmaco-miR: linking microRNAs and drug effects. Brief. Bioinform (2014) 15:648–59. doi: 10.1093/bib/bbs082 PMC4103536 23376192

[52] Jiang Q Wang Y Hao Y Juan L Teng M Zhang X. miR2Disease: a manually curated database for microRNA deregulation in human disease. Nucleic Acids Res (2009) 37:D98–104. doi: 10.1093/nar/gkn714 18927107 PMC2686559

[53] Ruepp A Kowarsch A Theis F. PhenomiR: microRNAs in human diseases and biological processes. Methods Mol Biol (2012) 822:249–60. doi: 10.1007/978-1-61779-427-8_17 22144204

[54] Yang J-H Li J-H Shao P Zhou H Chen Y-Q Qu L-H. starBase: a database for exploring microRNA-mRNA interaction maps from argonaute CLIP-seq and degradome-seq data. Nucleic Acids Res (2011) 39:D202–209. doi: 10.1093/nar/gkq1056 PMC3013664 21037263

[55] Wong N Wang X. miRDB: an online resource for microRNA target prediction and functional annotations. Nucleic Acids Res (2015) 43:D146–152. doi: 10.1093/nar/gku1104 PMC4383922 25378301

[56] Pedregosa F Varoquaux G Gramfort A Michel V Thirion B Grisel O. Scikit-learn: Machine learning in Python. J Mach Learn Res (2011) 12:2825–30.

[57] Chawla NV Bowyer KW Hall LO Kegelmeyer WP. SMOTE: Synthetic minority over-sampling technique. jair (2002) 16:321–57. doi: 10.1613/jair.953

[58] Al-Kafaji G Al-Muhtaresh HA Salem AH. Expression and clinical significance of miR-1 and miR-133 in pre-diabetes. BioMed Rep (2021) 14:33. doi: 10.3892/br.2021.1409 33585035 PMC7873585

[59] Zhao X Chen Z Zhou Z Li Y Wang Y Zhou Z. High-throughput sequencing of small RNAs and analysis of differentially expressed microRNAs associated with high-fat diet-induced hepatic insulin resistance in mice. Genes Nutr (2019) 14:6. doi: 10.1186/s12263-019-0630-1 30820263 PMC6379981

[60] Dahlmans D Houzelle A Jörgensen JA Phielix E Lindeboom L Hesselink MKC. Evaluation of muscle microRNA expression in relation to human peripheral insulin sensitivity: A cross-sectional study in metabolically distinct subject groups. Front Physiol (2017) 8:711. doi: 10.3389/fphys.2017.00711 28983252 PMC5613141

[61] Luo M Xu C Luo Y Wang G Wu J Wan Q. Circulating miR-103 family as potential biomarkers for type 2 diabetes through targeting CAV-1 and SFRP4. Acta Diabetol (2020) 57:309–22. doi: 10.1007/s00592-019-01430-6 31583475

[62] Kuleshov MV Jones MR Rouillard AD Fernandez NF Duan Q Wang Z. A Comprehensive Gene Set Enrichment Analysis Web Server 2016 Update. Nucleic Acids Res (2016) 44(W1):W90–7. doi: 10.1093/nar/gkw377 PMC4987924 27141961

[63] Xie Z Bailey A Kuleshov MV Clarke DJB Evangelista JE Jenkins SL. Gene Set Knowledge Discovery with Enrichr. Curr Protoc (2001) 1(3):e90. doi: 10.1002/cpz1.90 PMC8152575 33780170

[64] Karki R Kodamullil AT Hofmann-Apitius M. Comorbidity analysis between alzheimer’s disease and type 2 diabetes mellitus (T2DM) based on shared pathways and the role of T2DM drugs. J Alzheimers. Dis (2017) 60:721–31. doi: 10.3233/jad-170440 PMC5611890 28922161

[65] Zhou Y Deng J Chu X Zhao Y Guo Y. Role of post-transcriptional control of calpain by miR-124-3p in the development of alzheimer’s disease. J Alzheimer's Dis (2019) 67:571–81. doi: 10.3233/jad-181053 30584150

[66] Soares Bispo Santos Silva D Antunes J Balamurugan K Duncan G Sampaio Alho C McCord B. Evaluation of DNA methylation markers and their potential to predict human aging. Electrophoresis (2015) 36:1775–80. doi: 10.1002/elps.201500137 26010003

[67] Koch CM Wagner W. Epigenetic-aging-signature to determine age in different tissues. Aging (2011) 3:1018–27. doi: 10.18632/aging.100395 PMC3229965 22067257

[68] Mawlood SK Dennany L Watson N Pickard BS. The EpiTect methyl qPCR assay as novel age estimation method in forensic biology. Forensic Sci Int (2016) 264:132–8. doi: 10.1016/j.forsciint.2016.03.047 27108355

[69] van Steenoven I Koel-Simmelink MJA Vergouw LJM Tijms BM Piersma SR Pham TV. Identification of novel cerebrospinal fluid biomarker candidates for dementia with lewy bodies: A proteomic approach. Mol Neurodegener. (2020) 15:36. doi: 10.1186/s13024-020-00388-2 32552841 PMC7301448

[70] Lee S-J Wei M Zhang C Maxeiner S Pak C Calado Botelho S. Presynaptic neuronal pentraxin receptor organizes excitatory and inhibitory synapses. J Neurosci (2017) 37:1062–80. doi: 10.1523/JNEUROSCI.2768-16.2016 PMC5296791 27986928

[71] Mariga A Glaser J Mathias L Xu D Xiao M Worley P. Definition of a bidirectional activity-dependent pathway involving BDNF and narp. Cell Rep (2015) 13:1747–56. doi: 10.1016/j.celrep.2015.10.064 PMC4681298 26655895

[72] Tonne JM Sakuma T Deeds MC Munoz-Gomez M Barry MA Kudva YC. Global gene expression profiling of pancreatic islets in mice during streptozotocin-induced β-cell damage and pancreatic glp-1 gene therapy. Dis Model Mech (2013) 6:1236–45. doi: 10.1242/dmm.012591 PMC3759343 23828045

[73] Moran LB Hickey L Michael GJ Derkacs M Christian LM Kalaitzakis ME. Neuronal pentraxin II is highly upregulated in parkinson’s disease and a novel component of lewy bodies. Acta Neuropathologica (2008) 115:471–8. doi: 10.1007/s00401-007-0309-3 PMC2270353 17987278

[74] Sakharkar MK Kashmir Singh SK Rajamanickam K Mohamed Essa M Yang J Chidambaram SB. A systems biology approach towards the identification of candidate therapeutic genes and potential biomarkers for parkinson's disease. PloS One (2019) 14:e0220995. doi: 10.1371/journal.pone.0220995 31487305 PMC6728017

[75] Lang Y Li Y Yu H Lin L Chen X Wang S. HOTAIR drives autophagy in midbrain dopaminergic neurons in the substantia nigra compacta in a mouse model of parkinson’s disease by elevating NPTX2 *via* miR-221-3p binding. Aging (2020) 12:7660–78. doi: 10.18632/aging.103028 PMC7244061 32396526

[76] Xu C Tian G Jiang C Xue H Kuerbanjiang M Sun L. NPTX2 promotes colorectal cancer growth and liver metastasis by the activation of the canonical wnt/β-catenin pathway *via* FZD6. Cell Death Dis (2019) 10:217. doi: 10.1038/s41419-019-1467-7 30833544 PMC6399240

[77] Vatandoost N Amini M Iraj B Momenzadeh S Salehi R. Dysregulated miR-103 and miR-143 expression in peripheral blood mononuclear cells from induced prediabetes and type 2 diabetes rats. Gene (2015) 572:95–100. doi: 10.1016/j.gene.2015.07.015 26164754

[78] Trajkovski M Hausser J Soutschek J Bhat B Akin A Zavolan M. MicroRNAs 103 and 107 regulate insulin sensitivity. Nature (2011) 474:649–53. doi: 10.1038/nature10112 21654750

[79] Favereaux A Thoumine O Bouali-Benazzouz R Roques V Papon M-A Salam SA. Bidirectional integrative regulation of Cav1.2 calcium channel by microRNA miR-103: role in pain. EMBO J (2011) 30:3830–41. doi: 10.1038/emboj.2011.249 PMC3173784 21804529

